# Effect of Polymer Drag Reducer on Rheological Properties of Rocket Kerosene Solutions

**DOI:** 10.3390/ma15093343

**Published:** 2022-05-06

**Authors:** Xiaodie Guo, Xuejiao Chen, Wenjing Zhou, Jinjia Wei

**Affiliations:** 1 School of Chemical Engineering and Technology, Xi’an Jiaotong University, Xi’an 710049, China; gxd0915@stu.xjtu.edu.cn (X.G.); jjwei@xjtu.edu.cn (J.W.); 2 Beijing Institute of Aerospace Testing Technology, Beijing 100074, China; xuejiaocup@163.com; 3 State Key Laboratory of Multiphase Flow in Power Engineering, Xi’an Jiaotong University, Xi’an 710049, China

**Keywords:** drag reduction agent, rocket kerosene, rheological properties, molecular dynamics

## Abstract

Adding drag reduction agent (DRA) to rocket kerosene is an effective way to reduce the pipeline resistance of rocket kerosene transportation systems. However, so far, there have been few research reports on the effect of DRA on the rheological properties of rocket kerosene solution, especially from a microscopic perspective. In this study, coarse-grained molecular dynamics simulations were conducted to investigate the rheological properties of rocket kerosene solutions with DRAs of different chain lengths and concentrations. The results showed that the viscosity of DRA—kerosene solution is generally higher than that of pure kerosene at a low shear rate, while with an increase in shear rate, the viscosity of DRA—kerosene solution decreases rapidly and finally tends to become similar to that of pure kerosene. The shear viscosity of DRA—kerosene solution increases with an increase in chain length and concentration of polymers. Through observing the morphologic change of DRA molecules and analyzing the radius of gyration and the mean-squared end-to-end distance of polymers, it was confirmed that the rheological properties of DRA—kerosene solutions are strongly related to the degree of entanglement of polymer chains. The simulation results provide microscopic insights into the rheological behavior of DRA—kerosene solutions and clarify the intrinsic relation between the morphologic change of polymer molecules and the rheological properties of DRA—kerosene solutions.

## 1. Introduction

The development of manned spaceflight, manned lunar landings, Mars exploration, and other major space activities requires the support of advanced rocket engines. According to the development trend for liquid propellant rocket engines [1,2,3], large-thrust liquid oxygen/kerosene engines and heavy carrier rockets will be the major design goals for the future. However, due to the increase in propellant flow rate and the influence of extreme heat flow, it is inevitable that pipeline resistance will increase greatly and that pump load will be large. Turbo pump power and generator temperature will greatly increase, which will affect the reliability of engines, limiting engine performance improvement [4]. Therefore, it is necessary to reduce the frictional resistance of rocket kerosene in the pipeline. Adding polymer additives to turbulent flow is a well-known method of hydrodynamic drag reduction.

The drag reduction effect was first observed by Tomes [5] in 1948, when he dissolved a small amount of polymer (polymethyl methacrylate) in monochlorobenzene and found that the flow pressure in turbulent pipes was significantly reduced compared with pure solvents. Similar observations were subsequently made in other scholars’ studies. For example, Virk’s research has shown that in turbulent boundary layers, dissolving one part per million of a long-chain flexible polymer in a solution can reduce turbulent friction losses by up to 80% compared to using the solvent alone [6]. This attracted wide attention and led to extensive research on drag reduction. 

At present, the application of drag reduction agents in crude oil and refined oil pipeline transportation is very mature, but the drag reduction of rocket kerosene transportation systems is still in the stage of experimental demonstration. Lee et al. [7] investigated the turbulent drag reduction effect of the oil-soluble polymer polyisobutene in kerosene and explored the influence of molecular weight, Reynolds number (*Re*), and temperature on the time-dependence of drag reduction and explained the drag reduction phenomenon theoretically. Du et al. [8] conducted an experimental study on the flow resistance characteristics of rocket kerosene in pipelines and investigated the influence of various factors, such as the type of drag reduction agent (DRA), pipeline diameter, fluid flow rate, and concentration of DRA, on flow resistance. The results showed that flow resistance in the pipeline could be greatly reduced after adding DRA into the rocket kerosene and that the drag reduction efficiency could reach up to 75.05%. Zhang et al. [4] subsequently explored the drag reduction effect of DRA on high-temperature rocket kerosene under supercritical pressure and found that the drag reduction rate of drag reducers on rocket kerosene is affected by fluid temperature and *Re* and that the drag reduction rate of DRA could reach up to 60%. Li et al. [9] studied the drag reduction effect and heat transfer performance of supercritical kerosene with the addition of polymer additives by conducting CFD numerical simulations. The results showed that the pressure drop and heat transfer coefficient of supercritical kerosene flow was reduced by 46.8% and 37.5%, respectively.

Although the drag reduction effect of DRA has been studied experimentally and numerically, the mechanism of reducing the flow resistance of rocket kerosene by DRA is still unclear. The main reason is that it is still difficult to understand the interaction between the DRA molecules and the flow field. Therefore, in this paper, we study the effect of DRA in rocket kerosene from two aspects: rheological properties and molecular structure. From the macroscopic perspective, the rheological properties of rocket kerosene play an important role in hydrodynamic performance and drag reduction, while from the microscopic perspective, the morphologic change of the molecular structure of DRA is closely related to the flow field and energy transfer. In recent decades, a molecular dynamics (MD) method has been widely used to study the rheological properties of dilute polymer solutions [10,11,12,13,14,15], so we use an MD method to conduct simulations of rocket kerosene in this study. By studying the change in rheological properties of rocket kerosene before and after adding DRA and its relationship with the microstructure of DRA molecules, we can better understand the drag reduction mechanism of rocket kerosene by adding DRA, evaluate the performance of DRA in rocket kerosene, and then guide the practical application in engineering. 

The paper is organized as follows. The model construction and simulation methods are described in Section 2. The rheological properties of the system are analyzed in the first part of Section 3 and the morphologic change in the molecular structure of DRA and related explanations are given in the remaining parts of the section. Finally, a summary of the research results is given in Section 4.

## 2. Materials and Methods

### 2.1. System Model

Rocket kerosene is a mixture of hydrocarbons with a boiling point between 150–300 °C which includes hundreds to thousands of components, such as alkanes, naphthenes, and aromatic hydrocarbons [16]. Due to the complexity of rocket kerosene components, it is difficult to directly construct a MD model containing all the components of rocket kerosene and the effective method is to select several representative components from the components to build a simplified model [17]. In this study, a rocket kerosene with a known distribution of carbon mass fraction was analyzed and it was found that alkane, cycloalkane, and bicycloalkane accounted for 95.4% of the total mass fraction of rocket kerosene, so only these three components were considered in the simulation. The average carbon numbers of the three components were calculated to be 13, 13, and 12, respectively. Based on the average carbon number of these three substances and the most common rocket kerosene composition in the literature, n-tridecane [18], n-heptylcyclohexane [19], and naphthalene, decahydro-2,6-dimethyl [20,21], were selected as the representative components of rocket kerosene in this paper. Each component was added according to its mass percentage. The number of molecules and atoms of each component is shown in Table 1.

**Figure 1:**
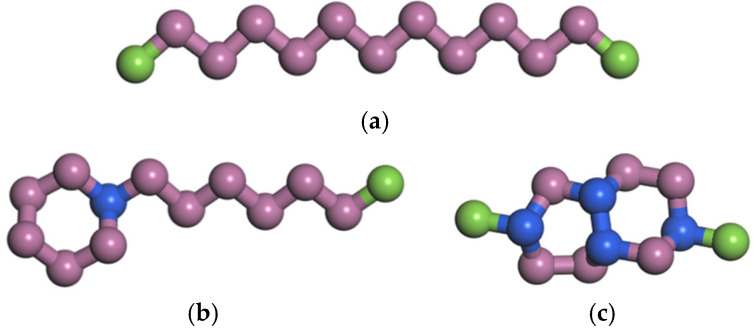
The molecular structure of (**a**) n-tridecane, (**b**) n-heptylcyclohexane, and (**c**) naphthalene, decahydro-2,6-dimethyl- in rocket kerosene. The green, pink and blue beads represent the CH3, CH2 and CH pseudoatomic groups, respectively.

**Figure 2:**
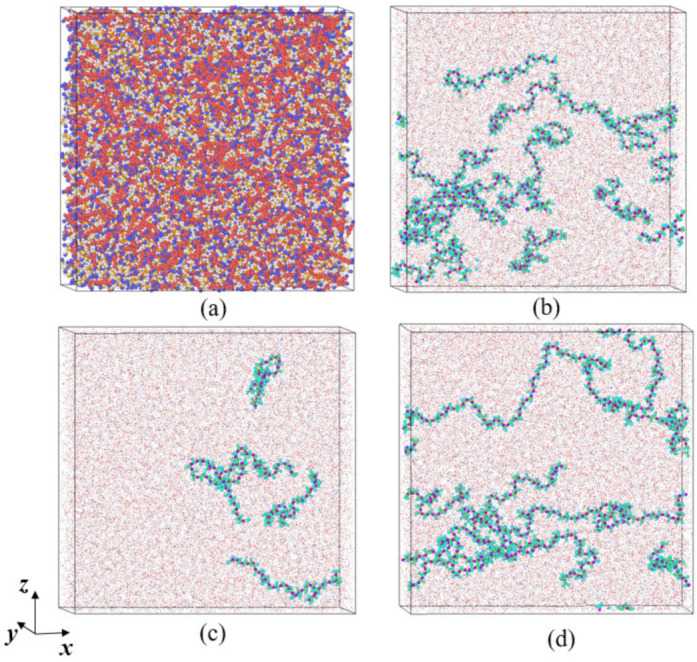
Snapshots of simulation systems: (**a**) pure kerosene; (**b**) DRA-kerosene solution ns36np15 with *N*_S_ = 36, *N*_p_ = 15, and *ϕ* = 0.086; (**c**) DRA-kerosene solution ns36np5 with *N*_S_ = 36, *N*_p_ = 5, and *ϕ* = 0.0287; (**d**) DRA-kerosene solution ns60np9 with *N*_S_ = 60, *N*_p_ = 9, and *ϕ* = 0.086.

In order to simplify the simulation model and save computational resources, the Transferable Potentials for Phase Equilibria-United Atom (TraPPE-UA) force field [22] was used. Compared with other force fields, TraPPE-UA can well reproduce liquid properties, especially liquid density [23,24,25]. The idea of the Trappe-UA force field is to unite each carbon and its bonded hydrogen into a single interaction site. According to this idea, the molecular structure of each component is shown in Figure 1. Experimental studies have shown that polyisobutene used in combustion chamber cooling channels can significantly reduce pressure loss [26], so polyisobutene was selected as the drag reduction agent (DRA). The polymer chains were composed of *N*S monomers. At the initial stage, *N*p polymer chains were randomly distributed in the simulation box. Two polymer chains with different lengths were considered: *N*S = 36 and *N*S = 60. For the short-chain polymer, i.e., *N*S = 36, two different mole fractions were considered: *ϕ* = 0.0287 and *ϕ* = 0.086. The corresponding polymer chain numbers were *N*p = 5 and *N*p = 15, respectively. For convenience, these two solutions are denoted as ns36np5 and ns36np15. For the polymer of *N*S = 60, the polymer concentration was set to be *ϕ* = 0.086 and the corresponding polymer chain number was *N*p = 9, denoted as ns60np9. The initial size of the simulation box was determined to be 140 Å × 140 Å × 140 Å. The studies of Packmol [27] and Moltemplate [28] were used to construct the kerosene and the DRA–kerosene solutions. A snapshot of the simulation system is shown in Figure 2.

### 2.2. TraPPE-UA Force Field

A TraPPE-UA force field was used to conduct the MD simulations and its potential function is expressed as follows [29]:

(1) $$E_{total}=E_{nonbond}+E_{stretch}+E_{angle}+E_{torsion}$$

where *E_total_*, *E_nonbond_*, *E_stretch_*, *E_angle_*, and *E_torsion_* represent total potential energy, non-bonded potential energy, bond-stretching potential energy, bond-angle bending potential energy, and torsional potential energy, respectively. Non-bonded interactions, which represent interactions between pseudoatoms separated by more than three bonds or belonging to different molecules, can be described by pairwise-additive Lennard-Jones 12-6 potentials:

(2) $$E_{nonbond}=\sum_{i,j}4\varepsilon_{ij}\left({\left(\frac{\sigma_{ij}}{r_{ij}}\right)}^{12}-{\left(\frac{\sigma_{ij}}{r_{ij}}\right)}^{6}\right)$$

where *r_ij_* is the distance between atoms *i* and *j* and *ε_ij_* and *σ_ij_* represent the potential well depth and equilibrium distance between atoms, respectively. The LJ interaction parameters are given in Table 2. The standard Lorentz–Berthelot combination rules are adopted for the cross parameters of the Lennard-Jones interaction between different pseudoatoms.

(3) $$\sigma_{ij}=\left(\sigma_{ii}+\sigma_{jj}\right)/2$$

(4) $$\varepsilon_{ij}={\left(\varepsilon_{ii}\varepsilon_{jj}\right)}^{1/2}$$

In the TraPPE-UA force field, pseud-atoms are connected by fixed bond lengths (all bond lengths are 1.54 Å). In order to preserve the flexibility of the molecule, bond stretching was considered. The bond connecting two atoms is described by the harmonic bond-stretching potential:

(5) $$E_{stretch}=\frac{1}{2}K{\left(R-R_{0}\right)}^{2}$$

where *K* is the elastic constant of bond stretching, *R* is the distance between adjacent particles, and *R*_0_ is the equilibrium bond length. The parameters of bond-stretching potential energy are shown in Table 3.

Two adjacent bonds form a bond angle *θ* whose motion is governed by a harmonic potential:

(6) $$E_{angle}=\frac{1}{2}K_{\theta }{\left(\theta -\theta_{0}\right)}^{2}$$

where *θ*, *θ*_0_, and *K_θ_* are the measured bending angle, the equilibrium bending angle, and the force constant, respectively. The values of *θ*_0_ and *K_θ_* are listed in Table 4.

Four interconnected particles in the molecule form a dihedral angle, and the dihedral angle constantly twists in the simulation process. The potential energy that describes this torsion is the torsional potential energy, and its potential energy function is expressed as follows:

(7) $$E_{torsion}=C_{0}+C_{1}\left(1+\cos\left(\varphi \right)\right)+C_{2}\left(1-\cos\left(2\varphi \right)\right)+C_{3}\left(1+\cos\left(3\varphi \right)\right)$$

where φ is the dihedral angle and $C_{i=0:3}$ are the Fourier coefficients. Values of these coefficients are given in Table 5. It should be noted that the parameters of bond stretching, bending angle, and dihedral angle torsion of branches in monocycloalkanes and dicycloalkanes are consistent with those of linear chains with the same group. For the sake of distinction, pseudoatoms on normal alkanes are represented by CH*_x_*, and pseudoatoms on cycloalkanes are represented by CH*_x_*_(cyc)_. 

### 2.3. Simulation Details

In this paper, the RNEMD method (also known as Müller-Plathe method) proposed by Müller-Plathe [33] was used to calculate shear viscosity. The core idea of this method is to obtain shear viscosity by applying momentum flux, which is difficult to measure, and calculating the shear rate or velocity profile, which are easy to measure. The calculation formulas involved are shown in Equations (8) and (9):

(8) $$j_{y}\left(p_{x}\right)=-\eta \frac{\partial v_{x}}{\partial y}$$

(9) $$j_{y}\left(p_{x}\right)=\frac{p_{\mathrm{total}}}{2tL_{x}L_{z}}$$

where $\frac{\partial v_{x}}{\partial y}$ is the gradient of the velocity in the *x* direction along the *y* direction (also known as the shear rate). The momentum flux *j_y_*(*P_x_*) is the *x* component of momentum transported in the *y* direction per unit area at a given time. The proportional coefficient between shear rate and transverse linear momentum flux is shear viscosity *η*. *P*_total_ is the accumulation of momentum exchange in the *x* direction, *t* is the total simulation time, *L_x_* and *L_z_* represent the length of the simulation box perpendicular to the direction of momentum flux. Due to the periodic boundary conditions of the system, the coefficient 2 in Equation (9) is required.

According to the idea of the method, the simulation box is divided into *M* slabs along the *y* direction and then a momentum flux is artificially imposed along the *y* direction on the system. The process is as follows: firstly, find out the atom in the first slab with the largest momentum component in the −*x* direction and the atom in the (*M*/2) + 1 slab with the largest momentum component in the +*x* direction; secondly, exchange the *x* components of the momentum between the two atoms with the same mass and perform a momentum exchange every *N* time steps. As the simulation proceeds, this will produce a velocity profile in the simulation box. The shear rate is obtained by calculating the slope of the velocity profile, then the shear viscosity at a given shear rate is calculated according to Equation (8). 

In this study, the simulation box was divided into 20 slabs along the *y* direction. During the simulation, the shear rate was controlled by adjusting the momentum exchange interval *N*. The simulation process of RNEMD was carried out at 293 K and 0.1 MPa. The whole simulation process was divided into two stages. In the first stage, the energy minimization process was performed and then a relaxation process of 3 ns was carried out in the isothermal and isobaric (NPT) ensemble to obtain a reasonable initial simulation configuration. The Nosé–Hoover method [34,35] was used for controlling the temperature and pressure of the system. The second stage involved data collection, including velocity gradient and momentum flux. The momentum exchange intervals were set to be 10, 20, 50, 100 and 200. The simulation time of this stage for the pure kerosene and the DRA–kerosene solution was 6 ns and 32 ns, respectively. For all simulation processes, the time step was 1fs. The trajectories of molecules were recorded every 20 ps. The snapshots of trajectories were visualized by OVITO [36]. All simulations were performed in LAMMPS (Large-Scale Atomic/Molecular Massively Parallel Simulator) [37].

## 3. Results and Discussion

### 3.1. Shear Viscosity of Different Solutions

**Figure 3:**
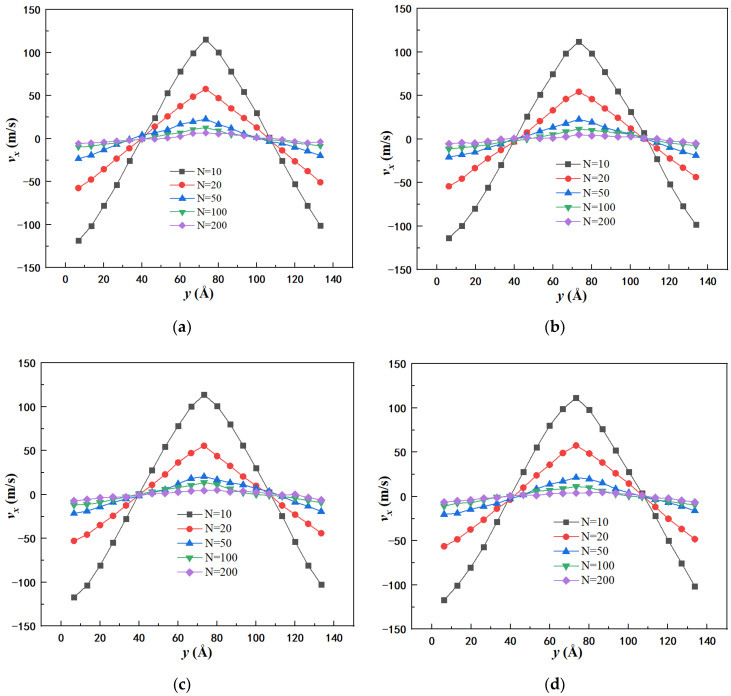
Velocity profiles in the *x* direction at momentum exchange intervals *N* = 10, 20, 50, 100, and 200 for: (**a**) pure kerosene; (**b**) ns36np15 solution; (**c**) ns36np5 solution; (**d**) ns60np9 solution.

In order to obtain the rheological characteristics and shear viscosity of DRA–kerosene solutions, the flow field of different shear rates was induced to pure kerosene and DRA–kerosene solutions by choosing different momentum exchange intervals. Figure 3 shows the velocity profile generated in the simulation box under different momentum exchange intervals. It can be seen that as momentum exchange interval decreases, velocity gradient and shear rate imposed on the simulation system increase. According to the velocity profile in Figure 3, the range of the magnitude of shear rate was found to lie between 1.0 × 10^9^ and 3.5 × 10^10^ s^−1^. 

**Figure 4:**
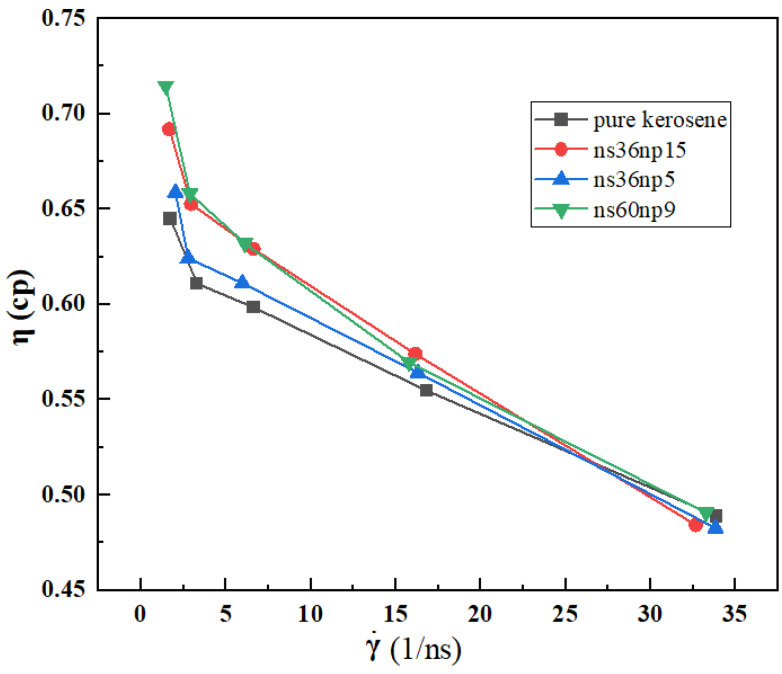
Dependence of viscosity η on shear rate $\dot{\gamma }$ for pure kerosene and DRA—kerosene solutions.

The shear viscosities at different shear rates for different systems are shown in Figure 4. As can be observed from the figure, with the increase in shear rate, the viscosities of both the pure kerosene and the DRA—kerosene solutions decrease significantly, resulting in the phenomenon of shear thinning. The viscosities of the DRA—kerosene solutions are higher than that of the pure kerosene at low shear rates. With the increase in shear rate, the viscosities of the DRA—kerosene solutions decrease rapidly and finally approach that of the pure kerosene. A possible reason is that after adding DRA to the pure kerosene, the polymer chains appear to be bent and curled at a low shear rate and are prone to entangle with each other, which increases the resistance of polymer chains in the solutions and presents an increase in fluid viscosity. As the shear rate increases, the polymer chains are stretched along the flow direction and the entangled polymer chains undergo chain disentanglement [38] and the relevant flow resistance decreases rapidly, resulting in a decrease in viscosity of the solutions with the increase in shear rate.

It can also be seen from Figure 4 that shear viscosities of the four systems at low shear rates are listed in descending order as follows: ns60np9 > ns36np15 > ns36np5 > pure kerosene, which indicates that the viscosity of the DRA–kerosene solution increases with increasing polymer concentration. A possible reason is that when the polymer concentration of the solution is low, the polymer molecules are scattered in the solution and the intermolecular forces of polymers are weak; with the increase in polymer concentration, the distance between polymer molecules decreases, which increases their probability of contacting each other, along with the degree of entanglement, so that the hydrodynamic volume of polymer molecules increases and the viscosity of the solution increases [39,40,41]. Of all the solutions, ns60np9, with a higher molecular weight of polymer, has the greatest viscosity at low shear rates. This can be understood from two aspects. Firstly, the larger molecular weight of a polymer indicates that more segments are contained in a polymer chain, and the polymer chain is more likely to slip between the chains when moving. Therefore, more energy needs to be consumed during the shearing process to overcome the synergy between the chains [42,43]. Secondly, the increase in molecular weight of the polymer also enhances the probability of entanglement and thus leads to greater flow resistance and eventually increases the shear viscosity [44].

### 3.2. Microstructure Evolution of Polymer Chains in DRA—Kerosene Solutions 

**Figure 5:**
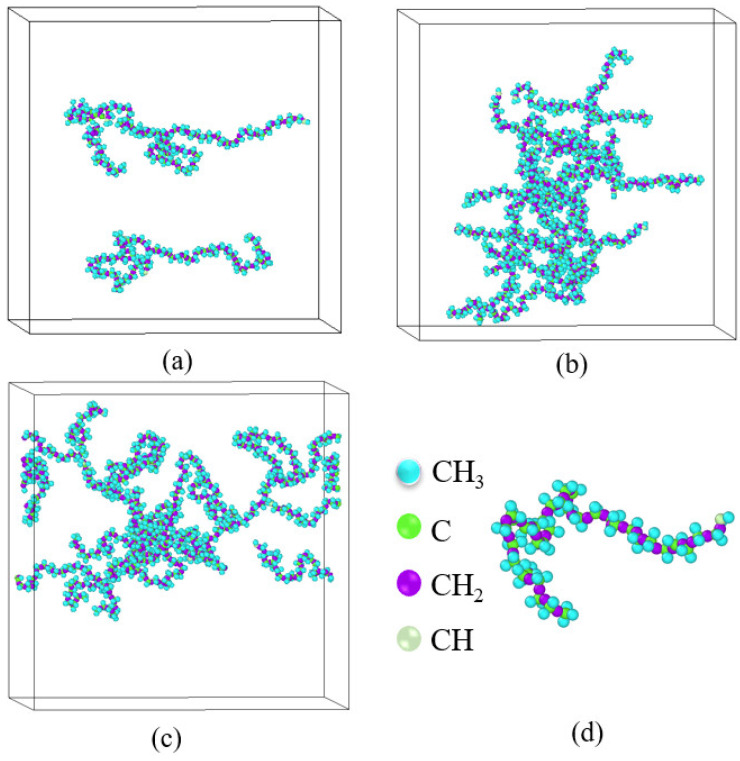
Conformational morphology of polymer molecules in (**a**) ns36np5, (**b**) ns36np15 and (**c**) ns60np9 solutions; (**d**) pseudoatomic groups in a single polymer molecule.

The conformational morphology of polymer molecules in the shearing process for ns36np5, ns36np15, and ns60np9 solutions at a low shear rate (*N* = 200) is shown in Figure 5. Different degrees of entanglement can be found in different solutions. In the ns36np5 solution, the polymer molecules were sparsely dispersed due to the small number of polymer chains. However, under the effect of Brownian motion of the polymer molecules, a slight entanglement of polymer molecules occurred in the shearing process. In the ns36np15 and ns60np9 solutions, due to the increase in the concentration and molecular weight of polymer chains, their chance of contacting each other was greatly increased, so the polymer chains tended to tangle with each other and form a network-like structure.

**Figure 6:**
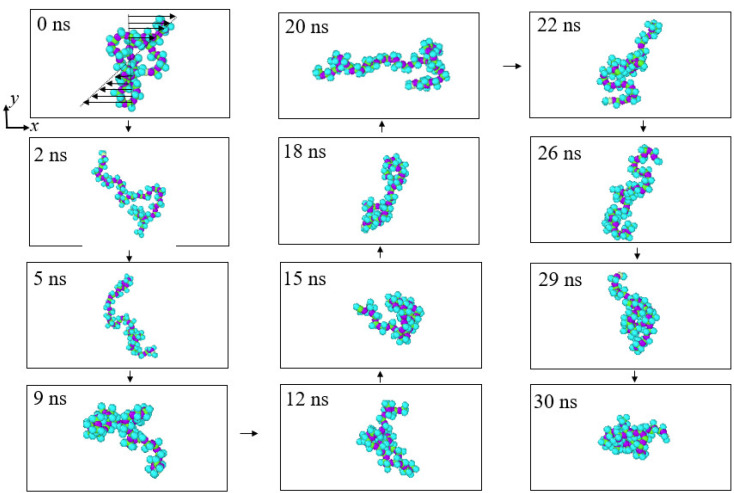
Snapshots of a polymer molecule in the ns36np15 solution at *N* = 200.

**Figure 7:**
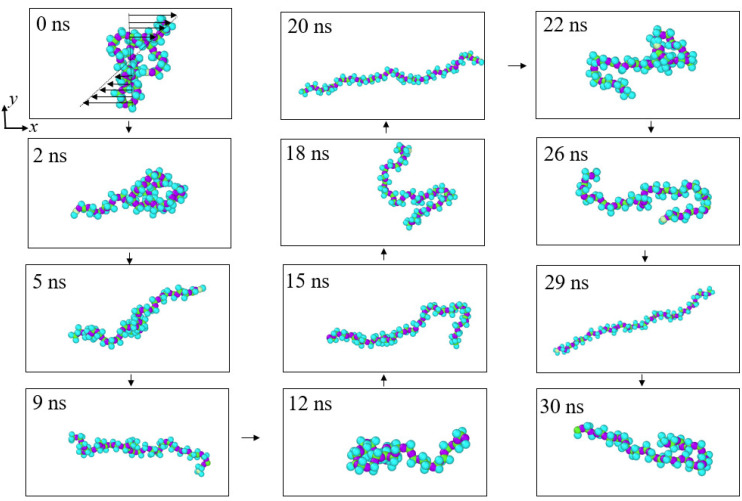
Snapshots of a polymer molecule in the ns36np15 solution at *N* = 10.

In order to offer an intuitive understanding of the conformational change of polymer molecules under the shearing effect, the evolution process of a single molecule in the ns36np15 solution with different shear rates corresponding to *N* = 200 and *N* = 10 is shown in Figure 6 and Figure 7, respectively. It can be seen that under the weak shearing flow (*N* = 200), the polymer molecule curls up into roughly ellipsoid form in most cases, while under the strong shearing flow (*N* = 10), the form of the polymer molecule alternates between curling and stretching states. Therefore, it can be considered that due to the presence of interactions between polymer molecules, entanglement between molecules will occur under low shear action and the degree of entanglement is enhanced with the increase in concentration and the molecular weight of polymers. With an increase in shear rate, the polymer molecules tend to stretch into elongated structures and change their orientation to the shearing direction, which destroys the entanglement of polymers, reduces flow resistance, and accordingly results in a decrease in the shear viscosity of solutions. Moreover, polymers in different solutions exhibit different stretching degrees under different shear rates, which will be introduced quantitatively in Section 3.3.

### 3.3. Quantitative Description of the Morphologic Change of Polymer Chains

In order to quantitatively compare the stretching degree of polymers under different shear rates, the radius of gyration and the mean-squared end-to-end distance of polymer molecules were calculated as follows:

(10) $$R_{g,i}^{2}=\frac{1}{M}{\sum m_{ia}\left({\vec{r}}_{ia}-{\vec{r}}_{cm,i}\right)}^{2}$$

(11) $$\langle R_{\mathrm{ete}}^{2}\rangle =\frac{1}{N_{p}}\frac{1}{N_{t}}{\sum_{i=1}^{N_{p}}\sum_{j=1}^{N_{t}}\left(r_{i,1}\left(j\right)-r_{i,n}\left(j\right)\right)}^{2}$$

where *M* is the molecular mass of a single polymer molecule and *m*_ia_ and ${\vec{r}}_{ia}$ represent the mass and position of the monomer *a* in the polymer molecule *i*, respectively. ${\vec{r}}_{cm,i}$ is defined as the center-of-mass position of the polymer molecule *i.* The radius of gyration *R*_g_ is the averaged calculation result for all polymer molecules in the system. *N*_p_ is the total number of polymer chains in solution. *N_t_* is the sampling times in the total simulation time. *r_i_*_,1_ and *r_i_*_,*n*_ represent the first atom and the last atom of the polymer molecule *i*, respectively. 

**Figure 8:**
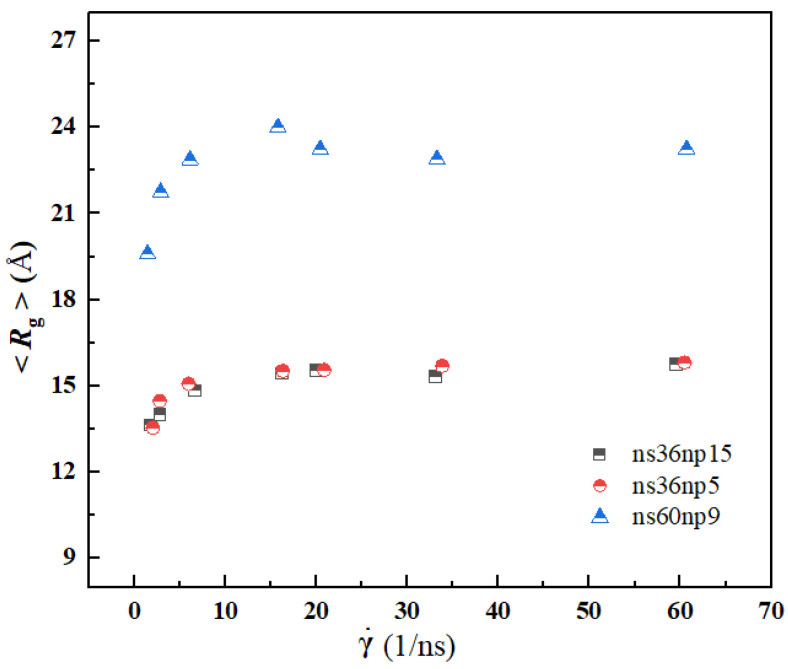
Variation of radius of gyration 〈*R_g_*〉 of polymers with shear rate.

**Figure 9:**
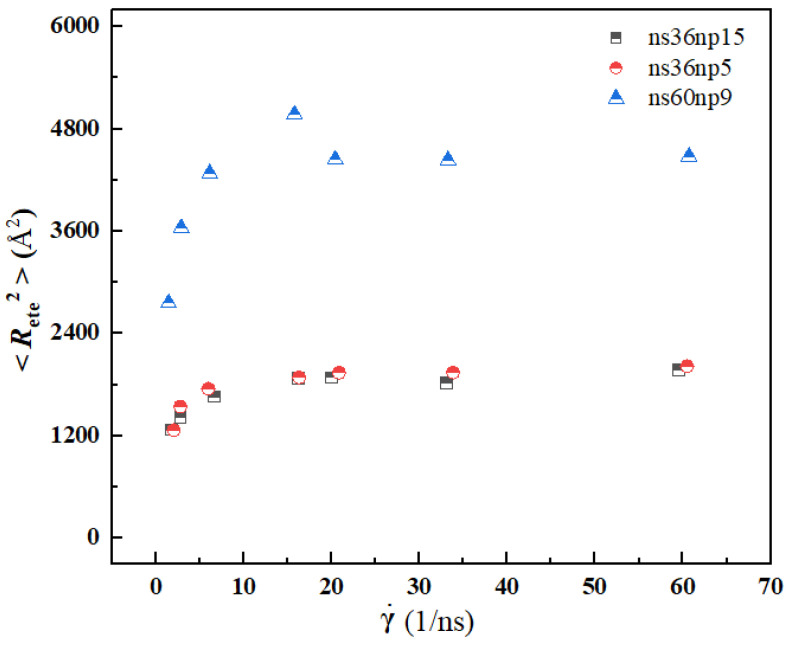
Variation of mean-squared end-to-end distance 〈*R*_ete_^2^〉 of polymers with shear rate.

Figure 8 and Figure 9 show the calculation results of radius of gyration 〈*R*_g_〉 and mean-squared end-to-end distance 〈*R*_ete_^2^〉 at different shear rates. It can be seen from the figure that with the increase in shear rate, the variation trend of 〈*R*_g_〉 and 〈*R*_ete_^2^〉 is similar, i.e., with the increase in shear rate, both of them increase sharply first and then reach a plateau, indicating that with the increase in shear rate, the curled and entangled polymer molecules in solutions are rapidly stretched. When the shear rate reaches a certain level, the entanglement between polymer chains is almost destroyed, and the extension degree of polymer chains reaches its limit. Due to a longer chain of polymers in the ns60np9 solution, the 〈*R*_g_〉 and 〈*R*_ete_^2^〉 of the ns60np9 solution are larger than those of the other two solutions.

**Figure 10:**
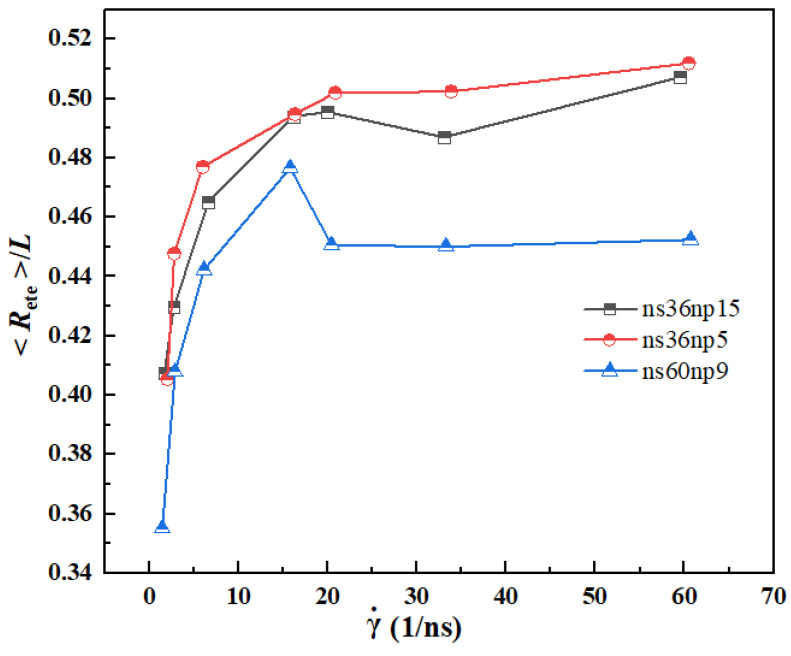
Variation of normalized root-mean-squared end-to-end distance 〈*R*_ete_〉/*L* of polymers with shear rate.

The variation of the normalized root-mean-squared end-to-end distance 〈*R*_ete_〉/*L* with shear rate for different solutions is plotted in Figure 10, where *L* represents the length of fully stretched polymers. It can be seen that for all three systems, with the increase in shear rate, 〈*R*_ete_〉/*L* increases sharply first and finally reaches a plateau. The value of the plateau region for the ns36np5 solution is close to 0.5, which is consistent with the results obtained by Xu et al. [38]. However, the value of the plateau region for the ns36np15 and ns60np9 solutions is slightly smaller, which is due to the fact that when the concentration or the chain length of polymers increase, the repulsive force between different polymer chains is stronger than that between different segments of the same polymer chain, resulting in incomplete stretching of polymers.

## 4. Conclusions

In this paper, the coarse-grained molecular dynamics method was used to study the rheological behavior of DRA—kerosene solutions and the conformational change of polymer molecules under steady shear flow conditions. The results show that DRA molecules in the solutions are easy to entangle and form network-like structures at low shear rates and that the degree of entanglement increases with the increase in the concentration and molecular weight of the polymer. This entanglement phenomenon increases the resistance to the motion of kerosene molecules, resulting in an increase in solution viscosity at low shear rates. With an increase in shear rate, the polymer molecules tend to be oriented along the flow direction and their molecular structure changes from a roughly ellipsoid form to a form alternating between curling and stretching, which is manifested by a sharp increase in the mean-squared end-to-end distance and the radius of gyration of polymers. At high shear rates, the network structures between polymer molecules are destroyed and the flow resistance decreases, so the shear viscosities of the solutions decrease greatly compared to those at low shear rates. With further increase in shear rate, the orientation of polymer chains is basically close to the limit state and the entanglement between molecules is completely destroyed. Correspondingly, the radius of gyration and the mean-squared end-to-end distance of polymers finally reach a plateau region. At this stage, the viscosities of DRA–kerosene solutions are consistent with that of pure rocket kerosene. The simulation results provide insight into the relation between the morphologic change of DRA molecules and the rheological properties of rocket kerosene solutions and have guiding significance for the application of DRA in aerospace working fluids. 

Future research should focus on the mechanism of drag reduction of rocket kerosene and the exploration of better molecular structures of DRA. Meanwhile, rocket kerosene also plays a role as a coolant in regenerative cooling engines, and the addition of DRA will affect the heat transfer capacity of rocket kerosene. Therefore, it is also important to study the relationship between drag reduction and heat transfer in DRA–kerosene solutions.

## Figures and Tables

**Table 1:** The number of molecules and atoms included in the rocket kerosene model.

Species	Components	Molecular Formula	Molecular Number	Atomic Number
Paraffins	n-tridecane	C13H28	1210	15,730
Monocycloalkanes	n-heptylcyclohexane	C13H26	2244	29,172
Dicycloalkanes	Naphthalene, decahydro-2,6-dimethyl	C12H22	2827	33,924
	sum	-	6281	78,826

**Table 2:** Lennard-Jones potential parameters for the TraPPE-UA force field.

United Atom Type	Molecule	*σ* (Å)	*ε*/*k*_B_ (K)
CH_3_ [22]	ALL	3.75	98
CH_2_ [22]	n-alkane/DRA	3.95	46
CH [30]	DRA	4.68	10
C [30]	DRA	6.4	0.5
CH_2(cyc)_ [31]	Monocylic and dicycloalkanes	3.91	52.5
CH_(cyc)_ [29]	Monocylic and dicycloalkanes	4.68	12

**Table 3:** Bond stretching parameters for the TraPPE-UA force field.

Bond Type	Molecule	*R*_0_ (Å)	*K* (kcal/mol/Å^2^)
CH*_x_*-CH*_y_* [32]	n-alkane/DRA	1.54	899.52
CH*_x_*_(cyc)_-CH*_y_*_(cyc)_ [29]	Monocylic and dicycloalkanes	1.54	536

**Table 4:** Bond angle parameters for the TraPPE-UA force field.

Bend Type	Molecule	*θ* (°)	*K*_θ_/*k*_B_ (K·rad^−2^)
CH*_x_*-CH_2_-CH*_y_* [22]	n-alkane/DRA	114	62,500
CH*_x_*_(cyc)_-CH_2(cyc)_-CH*_y_*_(cyc)_ [31]	Monocylic and dicycloalkanes	114	62,500
CH*_x_*_(cyc)_-CH_(cyc)_-CH_3_ (or CH_2_) [30]	Monocylic and dicycloalkanes	112	62,500
CH*_x_*-C-CH*_y_* [30]	DRA	109.47	62,500

**Table 5:** Dihedral torsion parameters for the TraPPE-UA force field.

Torsion	Molecule	*C*_0_/*k*_B_ (K)	*C*_1_/*k*_B_ (K)	*C*_2_/*k*_B_ (K)	*C*_3_/*k*_B_ (K)
CH*_x_*-(CH_2_)-(CH_2_)-CH*_y_* [22]	n-alkane	0	355.03	−68.19	791.32
CH_2(cyc)_-CH_2(cyc)_-CH_2(cyc)_-CH_(cyc)_ [29]	Monocylic and dicycloalkanes	0	355.03	−68.19	791.32
CH*_n_*_(cyc)_-CH_2(cyc)_-CH_(cyc)_-CH*_n_*_(cyc)_ (*n* = 1 or 2) [29]		−251.06	428.73	−111.85	441.27
CH*_x_*-(CH_2_)-(C)-CH*_y_* [30]	DRA	0	0	0	461.29

## Data Availability

Not applicable.

## References

[1] Cai H.H., Nie W.S., Guo K.K., Zhou S.Y. Influence of deflector on impact properties of multi-nozzle LOX/kerosene engine exhaust plume. Proceedings of the 2017 8th International Conference on Mechanical and Aerospace Engineering (ICMAE).

[2] Qin T., Rong Y., Qin X.D., Zhang Z. (2018). The development characteristics and trends of heavy launch vehicles. Aerosp. China.

[3] Sutton G.P. (2003). History of liquid propellant rocket engines in the United States. J. Propuls. Power.

[4] Zhang Z.J., Liu C.H., Pan H. (2019). Flow and heat transfer characteriatics of low-flow resistance rocket kerosene under supercritical pressure. J. Xi’an Jiaotong Univ..

[5] Toms B.A. Some observations on the flow of linear polymer solutions through straight tubes at large Reynolds numbers. Proceedings of the 1st International Congress on Rheology.

[6] Virk P.S. (1975). Drag reduction fundamentals. AIChE J..

[7] Lee K.H., Zhang K., Choi H.J. (2010). Time dependence of turbulent drag reduction efficiency of polyisobutylene in kerosene. J. Ind. Eng. Chem..

[8] Du Z.G., Zhu C.C., Wu J., Shan S.Q., Yu X.L., Shi X.M., Fu Q.J., Han W. (2017). investigation on drag-reduction technology of rocket kerosene. J. Rocket. Propuls..

[9] Li B., Li W.X., Zheng X., Wang Y., Tang M.M., Cai W.H. (2021). Numerical study on influences of drag reducing additive in supercritical flow of kerosene in a millichannel. Energies.

[10] Aust C., Kröger M., Hess S. (1999). Structure and dynamics of dilute polymer solutions under shear flow via nonequilibrium molecular dynamics. Macromolecules.

[11] Huang C.C., Gompper G., Winkler R.G. (2012). Non-equilibrium properties of semidilute polymer solutions under shear flow. J. Phys. Conf. Ser..

[12] Kong Y., Manke C.W., Madden W.G., Schlijper A.G. (1997). Modeling the rheology of polymer solutions by dissipative particle dynamics. Tribol. Lett..

[13] Prabhakar R., Prakash J.R. (2004). Multiplicative separation of the influences of excluded volume, hydrodynamic interactions and finite extensibility on the rheological properties of dilute polymer solutions. J. Non-Newton. Fluid Mech..

[14] Schlijper A.G., Hoogerbrugge P.J., Manke C.W. (1995). Computer simulation of dilute polymer solutions with the dissipative particle dynamics method. J. Rheol..

[15] Stoltz C., de Pablo J.J., Graham M.D. (2006). Concentration dependence of shear and extensional rheology of polymer solutions: Brownian dynamics simulations. J. Rheol..

[16] Edwards T. “Kerosene” fuels for aerospace propulsion-composition and properties. Proceedings of the 38th AIAA/ASME/SAE/ASEE Joint Propulsion Conference & Exhibit.

[17] Zheng D., Yu W.M., Zhong B.J. (2015). RP-3 aviation kerosene surrogate fuel and the chemical reaction kinetic model. Acta Phys. Chim. Sin..

[18] Farmer R.C., Anderson P.G., Cheng G.C. (1996). Propellant Chemistry for CFD Applications.

[19] Huber M.L., Lemmon E.W., Bruno T.J. (2009). Effect of RP-1 compositional variability on thermophysical properties. Energy Fuels.

[20] Zhang G.Y., Peng Q.T., Sheng T. (2011). GC-MS determination of components in rocket kerosene and rocket kerosene vapour. Missiles Space Veh..

[21] Kehimkar B., Hoggard J.C., Marney L.C., Billingsley M.C., Fraga C.G., Bruno T.J., Synovec R.E. (2014). Correlation of rocket propulsion fuel properties with chemical composition using comprehensive two-dimensional gas chromatography with time-of-flight mass spectrometry followed by partial least squares regression analysis. J. Chromatogr. A.

[22] Martin M.G., Siepmann J.I. (1998). Transferable potentials for phase equilibria. 1. United-atom description of n-alkanes. J. Phys. Chem. B.

[23] Martin M.G. (2006). Comparison of the AMBER, CHARMM, COMPASS, GROMOS, OPLS, TraPPE and UFF force fields for prediction of vapor–liquid coexistence curves and liquid densities. Fluid Phase Equilibria.

[24] Müller E.A., Mejia A. (2011). Comparison of united-atom potentials for the simulation of vapor–liquid equilibria and interfacial properties of long-chain n-alkanes up to n-c100. J. Phys. Chem. B.

[25] Papavasileiou K.D., Peristeras L.D., Bick A., Economou I.G. (2019). Molecular dynamics simulation of pure n-alkanes and their mixtures at elevated temperatures using atomistic and coarse-grained force fields. J. Phys. Chem. B.

[26] Borovik I., Strokach E., Kozlov A., Gaponov V., Chvanov V., Levochkin P., Yoon Y. (2019). Influence of polyisobutylene kerosene additive on combustion efficiency in a liquid propellant rocket engine. Aerospace.

[27] Martínez L., Andrade R., Birgin E.G., Martínez J.M. (2009). PACKMOL: A package for building initial configurations for molecular dynamics simulations. J. Comput. Chem..

[28] Jewett A.I., Stelter D., Lambert J., Saladi S.M., Roscioni O.M., Ricci M., Autin L., Maritan M., Bashusqeh S.M., Keyes T. (2021). Moltemplate: A tool for coarse-grained modeling of complex biological matter and soft condensed matter physics. J. Mol. Biol..

[29] Yiannourakou M., Ungerer P., Lachet V., Rousseau B., Teuler J.M. (2019). United atom forcefield for vapor-liquid equilibrium (VLE) properties of cyclic and polycyclic compounds from Monte Carlo simulations. Fluid Phase Equilibria.

[30] Martin M.G., Siepmann J.I. (1999). Novel configurational-bias Monte Carlo method for branched molecules. Transferable potentials for phase equilibria. 2. United-atom description of branched alkanes. J. Phys. Chem. B.

[31] Keasler S.J., Charan S.M., Wick C.D., Economou I.G., Siepmann J.I. (2012). Transferable potentials for phase equilibria-united atom description of five-and six-membered cyclic alkanes and ethers. J. Phys. Chem. B.

[32] Kioupis L.I., Maginn E.J. (1999). Rheology, dynamics, and structure of hydrocarbon blends: A molecular dynamics study of n-hexane/n-hexadecane mixtures. Chem. Eng. J..

[33] Müller-Plathe F. (1999). Reversing the perturbation in nonequilibrium molecular dynamics: An easy way to calculate the shear viscosity of fluids. Phys. Rev. E.

[34] Hoover W.G. (1985). Canonical dynamics: Equilibrium phase-space distributions. Phys. Rev. A.

[35] Nosé S. (1984). A unified formulation of the constant temperature molecular dynamics methods. J. Chem. Phys..

[36] Stukowski A. (2009). Visualization and analysis of atomistic simulation data with OVITO–the Open Visualization Tool. Modell. Simul. Mater. Sci. Eng..

[37] Plimpton S. (1995). Fast parallel algorithms for short-range molecular dynamics. J. Comput. Phys..

[38] Xu X.L., Chen J.Z., An L.J. (2014). Shear thinning behavior of linear polymer melts under shear flow via nonequilibrium molecular dynamics. J. Chem. Phys..

[39] Chan P.S., Chen J.S., Ettelaie R., Law Z., Alevisopoulos S., Day E., Smith S. (2007). Study of the shear and extensional rheology of casein, waxy maize starch and their mixtures. Food Hydrocoll..

[40] Nickzare M., Zohuriaan-Mehr M.J., Yousefi A.A., Ershad-Langroudi A. (2009). Novel acrylic-modified acacia gum thickener: Preparation, characterization and rheological properties. Starch-Stärke.

[41] Wang L.C., Wu Y.J., Li J.S., Qiao H.Z., Di L.Q. (2018). Rheological and mucoadhesive properties of polysaccharide from Bletilla striata with potential use in pharmaceutics as bio-adhesive excipient. Int. J. Biol. Macromol..

[42] Wang F., Ma Z.L., Du B., Li W., Wang D.P., Chen S.T., Wang Y.F. (2021). Study on tensile rheological properties of polyethylene melt. China Plast..

[43] Hou C., Wang C.G., Ying L. (2003). Effect of inorganic salts on viscosity of acrylonitrile-N-vinylpyrrolidone copolymer solutions. J. Appl. Polym. Sci..

[44] Devasia R., Nair C.P.R., Ninan K.N. (2008). Temperature and shear dependencies of rheology of poly (acrylonitrile-co-itaconic acid) dope in DMF. Polym. Adv. Technol..

